# Characterization of the complete mitochondrial genome of *Hynobius bambusicolus* Wang, Othman, Qiu and Borzée, 2023 (Amphibia, Caudata, Hynobiidae) and its phylogenetic implications

**DOI:** 10.1080/23802359.2025.2475845

**Published:** 2025-03-10

**Authors:** Yanpin Huang, Helin Wang, Haoran Luo, Honghui Zhong, Qingxian Lin, Xiaoping Zhou

**Affiliations:** a Key Laboratory of the Ministry of Education for Coastal and Wetland Ecosystems, College of the Environment and Ecology, Xiamen University, Xiamen, China; b Meihua Mountain National Nature Reserve Administration, Longyan, China

**Keywords:** Caudata, hynobidae, mitochondrial genome, high-throughput sequencing, phylogenetic analysis

## Abstract

*Hynobius bambusicolus* (Caudata, Hynobiidae) is a recently described species, identified in 2022, and is thus not widely known. In this study, we sequenced and annotated the complete mitogenome of *H. bambusicolus*. The resulting mitochondrial genome is 16,406 bp in length and comprises 13 protein-coding genes (PCGs), two ribosomal RNA genes (rRNA), 22 transfer RNA genes, and a non-coding region. The base composition of the mitogenome is 33.3% A, 32.1% T, 20.8% C, and 13.8% G. The phylogenetic trees indicated that *H. bambusicolus* is the basal branch within the Southern Chinese *Hynobius* clade.

## Introduction

**Figure 1.**
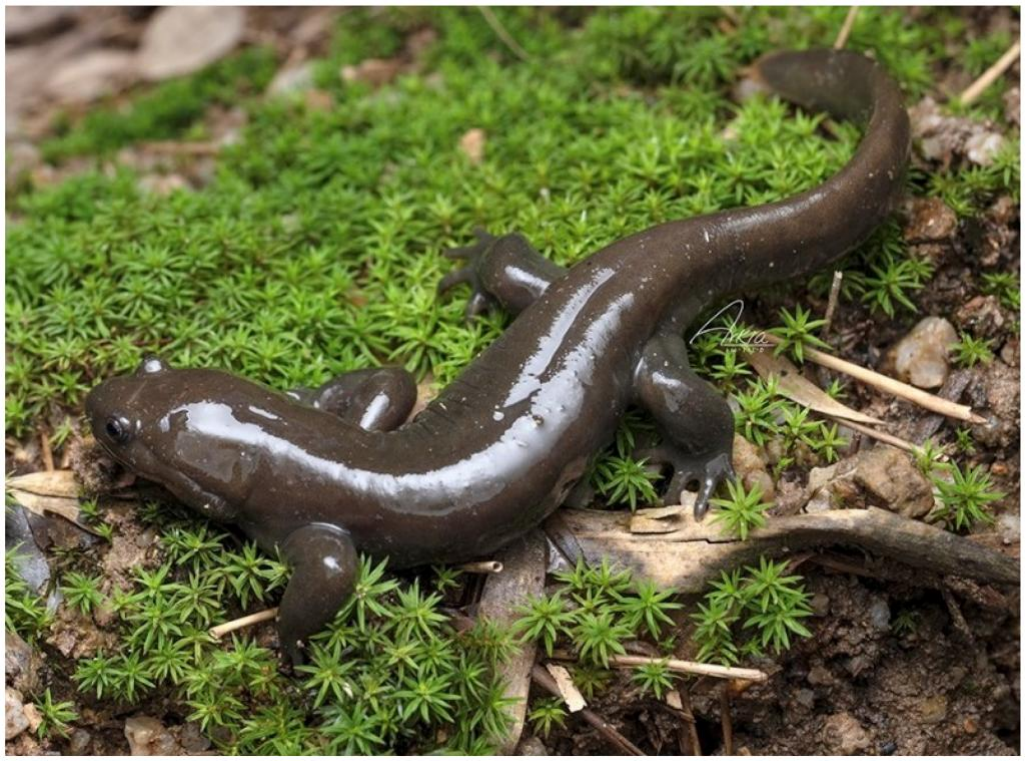
Reference image of the *Hynobius bambusicolus* (photographer: Zhenqi Wang, email: arkia0205@njfu.edu.cn, used with permission). This photograph was captured in April 2024. The individual in the figure is an adult with a total length of 17 cm.

*Hynobius* is the most diverse genus in Hynobiidae family, which includes 66 species (Frost 2024). To date, the complete mitochondrial genomes have only been sequenced for approximately 20 *Hynobius* species. *Hynobius bambusicolus* Wang, Othman, Qiu and Borzée, 2023 was first discovered by Wang et al. (2023) in January 2022 in Quxi village, Liancheng County, Fujian, China (Figure 1). Currently, this species is known from a single locality, with an estimated population size likely to be fewer than 200 breeding individuals, aligning with the Critically Endangered (CR) status recommended by the IUCN Red List of Threatened Species (Wang et al. 2023). The colonies of *H. bambusicolus* inhabit artificial bamboo forests, which are significantly threatened by anthropogenic activities (Wang et al. 2023). To date, the molecular data available for *H. bambusicolus* is limited to segments of a single mtDNA gene, including *rrnL*, *COX1*, and *CYTB* (Wang et al. 2023). Phylogenetic trees constructed using these single or concatenated mtDNA gene segments have produced incongruent results, leaving the phylogenetic position of this species unresolved. In this study, we present the first complete mitochondrial genome of *H. bambusicolus*, and infer its phylogenetic position within the genus *Hynobius* based on the concatenation of two ribosomal RNA genes (rRNA) genes and 13 protein-coding genes (PCGs).

## Materials and methods

### Sample collection, DNA extraction, and sequencing

A dead larva of *H. bambusicolus* was collected in April 2024 from Quxi village, Liancheng County, Fujian, China (25.566° N, 116.938° E) and was deposited at −80 °C in the laboratory at College of the Environment and Ecology, Xiamen University, Xiamen, China (https://cee.xmu.edu.cn/, Xiaoping zhou, xpzhou@xmu.edu.cn) under voucher number CEE2024Anura-Hb-01. The larva specimen was identified as *H. bambusicolus* based on its brown body and blue speckles on dorsum, as well as its sequences of *COX1* fragment 100.00% match to that reported for *H. bambusicolus* (GenBank accession number: OQ107447, Wang et al. 2023). A tail of the sample was sent to BGI Genomics Co., Ltd for DNA extraction and sequencing. DNA material was extracted using the Sodium Dodecyl Sulfate method (BGI Genomics Company Limited 2024). Genomic library was constructed using a BGI Optimal DNA Library Prep Kit (BGI-Shenzhen, China). The insert size was 300–400 bp. Sequencing was performed on the DNBSEQ platform using a pair-end sequencing protocol with a read length of 150 bp (PE150), and a total of 83,522,957 raw reads were yielded. Raw reads were filtered using SOAPnuke (Chen et al. 2018) to remove adapter sequences, contamination, and low-quality reads, which resulted in a total of 80,144,121 clean reads with the mean length of 150 bp.

### Mitogenome assembly and annotation

The mitochondrial genome was assembled using NOVOPlasty 4.3.5 software (Dierckxsens et al. 2017) with the first 150 bp of *COX1* (OQ107447) of *H. bambusicolus* (Wang et al. 2023) as a seed sequence. The coverage depth map, generated using the method described by Ni et al. (2023), showed an average depth of 220.31× (Supplementary Figure S1). The mitogenome was annotated using the MITOS (Bernt et al. 2013). The origin of L-strand replication (OL) and a non-coding region (putative D-loop) were identified by homology alignments with the corresponding known sequences of *H. dunni* Tago 1931 (GenBank accession number: LC538211, Igawa et al. 2020). The circular genome map was drawn using the CGView (Stothard and Wishart 2005).

### Phylogenetic analysis

Twenty mitogenome sequences of other *Hynobius* species were downloaded from GenBank to reconstruct phylogenetic trees alongside *H. bambusicolus*, with *Pachyhynobius shangchengensis* Fei et al. 1983 used as the outgroup (Table 1). The fifteen genes (*rrnS*, *rrnL*, and 13 PCGs) were aligned individually using ClustalW (Thompson et al. 1994) with default parameters and then concatenated into a single alignment for phylogenetic analysis. Maximum likelihood (ML) and Bayesian inference (BI) were used to construct phylogenetic trees, and the concatenated alignment dataset was partitioned by gene. The nucleotide substitution model of each locus (Supplementary Table S1) employed in the both ML and BI analysis was selected based on the Bayesian Information Criterion (BIC) *via* MEGA11 (Tamura et al. 2021). The ML analysis was conducted utilizing RAxML-NG (Kozlov et al. 2019) with 1000 bootstraps. The BI analysis was performed with MrBayes 3.2.7 (Ronquist et al. 2012) under the following parameters: 10,000,000 generations with a sampling frequency every 1000 generations, four MCMC chains, and a burn-in of 25%. The convergence was evaluated by a split frequency value lower than 0.01 and the value of the Estimated Sample Size (ESS) for each parameter higher than 200 calculated using tracer v1.7.2 (Rambaut et al. 2018). The phylogenetic trees were visualized using TreeViewer (Bianchini and Sánchez‐Baracaldo 2024).

**Table 1.** The information of the mitochondrial genome sequences of *Hynobius* and one outgroup used in the phylogenetic analysis.

Species	Accession number	Source	Localities
*Hynobius bambusicolus*	PQ220620	This study	Fujian, China
*Hynobius amjiensis*	DQ333808	Zhang et al. (2006)	Zhejiang, China
*Hynobius chinensis*	JQ710885	Unpublished	Southern China
*Hynobius guabangshanensis*	GU384690	Unpublished	Guabang Mountain, China
*Hynobius yiwuensis*	HM036354	Zheng et al. (2011)	Zheijiang, China
*Hynobius maoershanensis*	KF974475	Huang et al. (2016)	Guangzi, China
*Hynobius leechii*	DQ333811	Zhang et al. (2006)	Northeastern China
*Hynobius arisanensis*	EF462213	Unpublished	Central Taiwanese Island
*Hynobius formosanus*	DQ333816	Zhang et al. (2006)	Northerncentral Taiwanese Island
*Hynobius quelpaertensis*	EF201847	Unpublished	R. Korea
*Hynobius unisacculus*	MN419307	Unpublished	R. Korea
*Hynobius yangi*	FJ594965	Unpublished	R. Korea
*Hynobius nebulosus*	HM036356	Zheng et al. (2011)	Japan
*Hynobius kimurae*	JQ929920	Unpublished	Japan
*Hynobius nigrescens*	JQ929922	Unpublished	Japan
*Hynobius dunni*	LC538211	Igawa et al. (2020)	Japan
*Hynobius tsuensis*	JQ929923	Unpublished	Japan
*Hynobius hidamontanus*	JQ929919	Unpublished	Japan
*Hynobius tokyoensis*	HM036357	Zheng et al. (2011)	Japan
*Hynobius lichenatus*	JQ929921	Unpublished	Japan
*Hynobius retardatus*	HM036351	Zheng et al. (2011)	Japan
*Pachyhynobius shangchengensis*	MK890367	Pan et al. (2019)	Dabie Mountain, China

## Results

**Figure 2.**
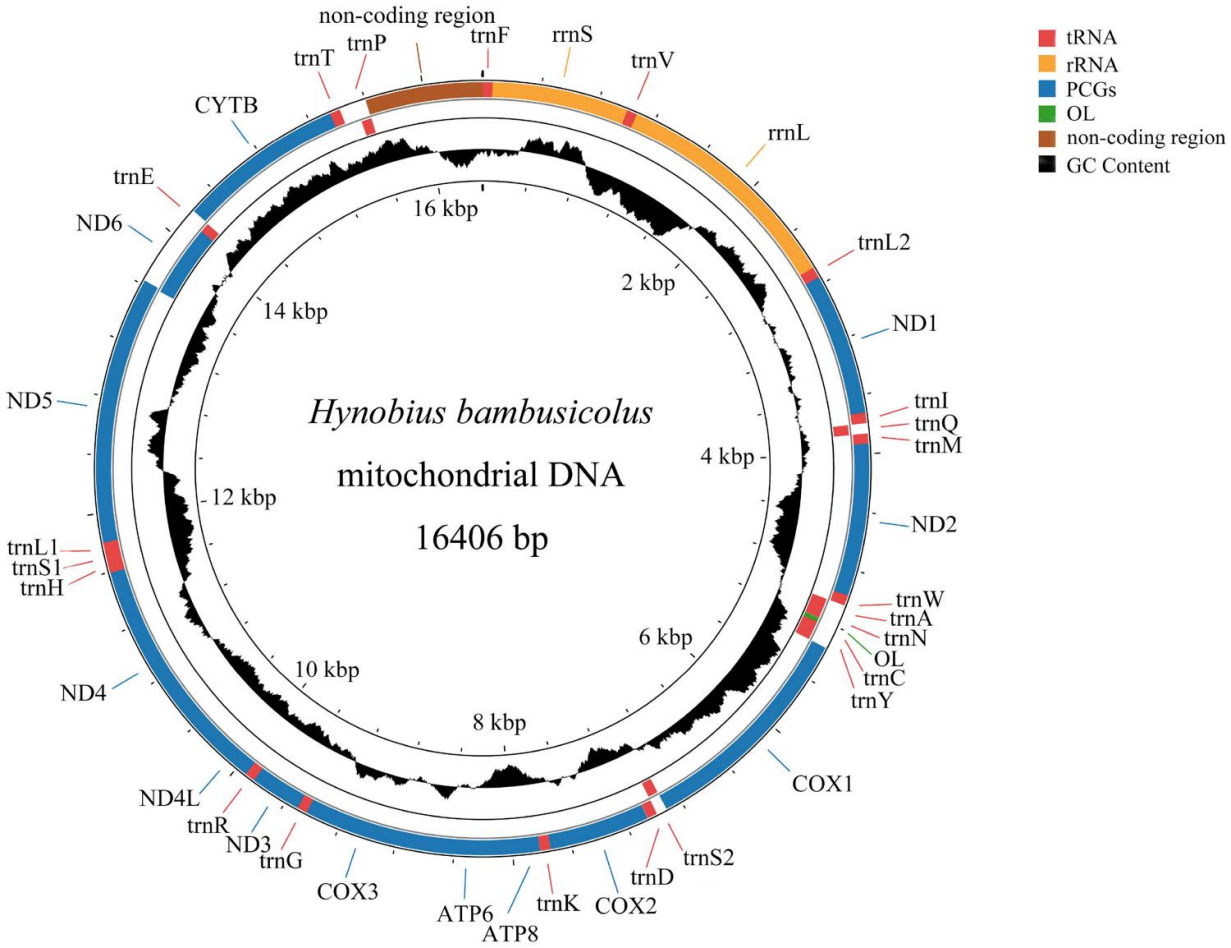
Circular map of the mitochondrial genome of *Hynobius bambusicolus*. Protein coding and ribosomal genes are shown with standard abbreviations. The genes in the inner are encoded on the L-strand. GC content plots are generated by calculating the GC content for each sliding window using the formula:G + C/Window Size, where values range between 0 and 1. The window moves by the step size, and the calculation repeats. The window size is set to 500 bp, and the step size is 1 bp. If the calculated value is higher than the GC content of the entire genome (0.346), it is displayed above the baseline; conversely, if it is lower, it is displayed below the baseline.

The complete mitogenome sequence of *H. bambusicolus* is 16,406 bp in length and comprises 13 PCGs, 22 transfer RNA genes (tRNA) genes, 2 rRNA genes (*rrnS* and *rrnL*), an origin of L-strand replication (OL), and a non-coding region (putative D-loop). With the exception of one PCG (*ND6*) and eight tRNAs *(trnQ*, *trnA*, *trnN*, *trnC*, *trnY*, *trnS2*, *trnE*, and *trnP*), the majority of genes are encoded by the heavy (H) strand (Figure 2). Furthermore, the nucleotide composition of *H. bambusicolus* mitogenome consists of 33.3% A, 32.1% T, 20.8% C, and 13.8% G, exhibiting a slight bias toward A + T (65.4%). *COX1* initiats with GTG, while the remaining 12 PCGs commence with ATG. The termination codons for the PCGs are as follows: TAA for *COX1*, *COX2*, *ATP8*, *ATP6*, *ND4L*, and *ND5*, AGA for *ND6*, incomplete stop codons TA for *ND1*, and incomplete stop codons T for *COX3*, *ND2*, *ND3*, *ND4*, and *CYTB*. The total length of tRNA genes is 1539 bp. The *rrnS* and *rrnL* in *H. bambusicolus* mitogenome are 935 bp and 1604 bp in length, respectively.

**Figure 3.**
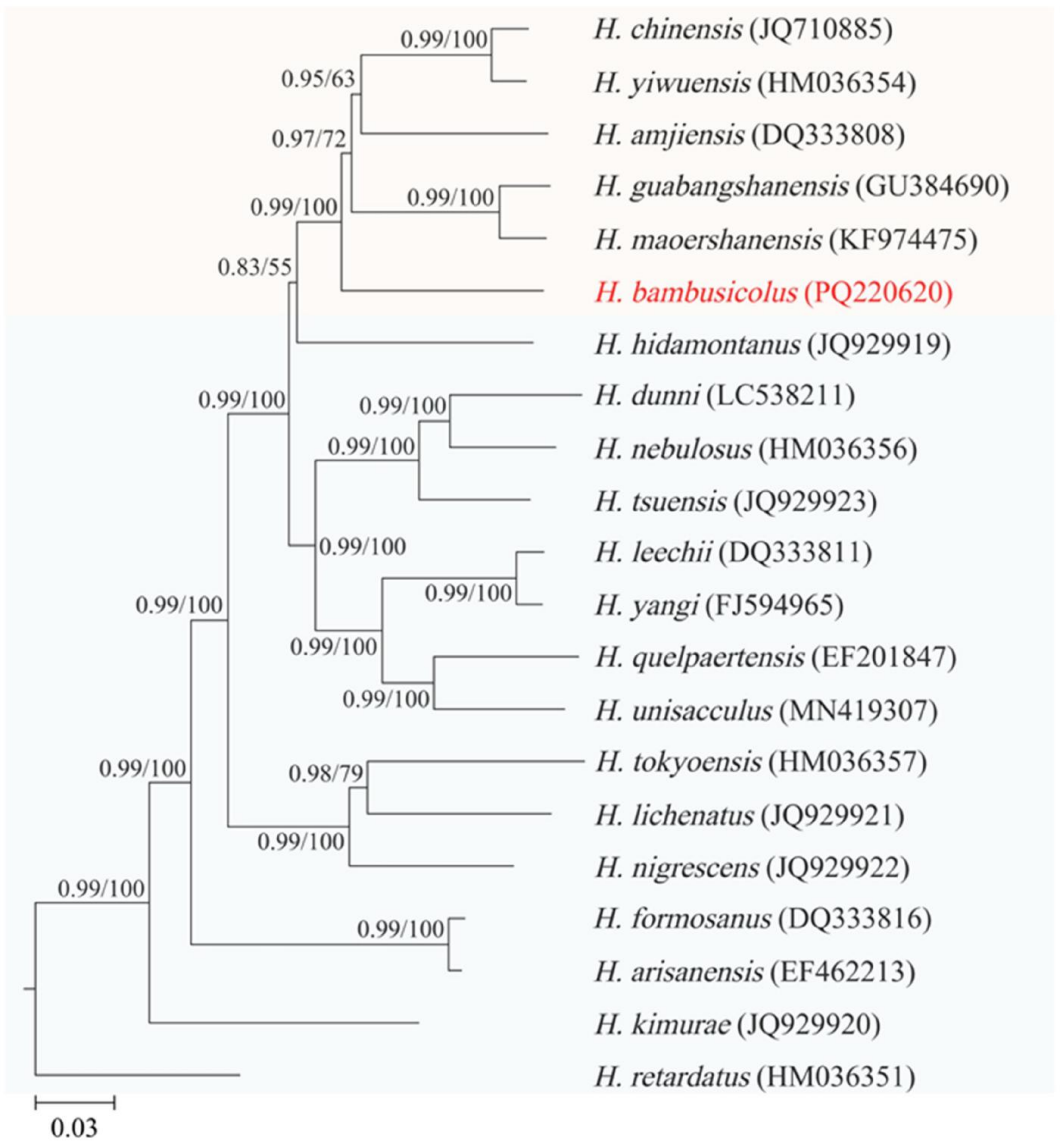
Phylogenetic tree for 21 *Hynobius* species distributed across East Asia based on the concatenated nucleotide sequences of *rrnS*, *rrnL*, and 13 PCGs. The outgroup taxa are not shown.The numbers on each node represent the Bayesian posterior probabilities (left) and ML bootstrap percentages (right). The number after the species name is the GenBank accession number. Orange background color represents Southern Chinese *Hynobius*, blue background color represents other *Hynobius*. Scale bar refers to a phylogenetic distance of 0.03 nucleotide substitutions per site.

The BI and ML phylogenetic trees had identical topologies and most nodes were supported by high posterior probabilities (PP) and bootstrap percentages (BP) (Figure 3). The results revealed that the southern Chinese *Hynobius* formed a monophyletic group, and the *H. bambusicolus* was the basal lineage sister to other species within this group.

## Discussion and conclusions

The gene composition and organization of the mitochondrial genome of *H. bambusicolus* were identical to those observed in other *Hynobius* species. The sequence length of complete mitochondrial genome of *Hynobius bambusicolus* was also comparable to those reported for other *Hynobius* species, which ranged from 16,394 bp in *Hynobius formosanus* Maki 1922 (Zhang et al. 2006) to 16,495 *bp* in *Hynobius chinensis* Günther 1889. The primary factor contributing to length variations in the mitochondrial genome of *Hynobiu*s species was the variation in the intergenic spacer between the *trnT* and *trnP*, which ranged from 113 bp in *Hynobius guabangshanensis* Shen et al., 2004 to 203 bp in *Hynobius yiwuensis* Cai 1985 and *H. chinensis*.

The results of phylogenetic analysis in this study supported the monophyly of the southern Chinese *Hynobius* clade and the basal position of *H. bambusicolus* within this clade (Wang et al. 2023). The internal topology of the southern Chinese *Hynobius* clade was consistent with the previous findings of Igawa et al. (2020), but not with the findings of Li et al. (2011). In our results, *Hynobius maoershanensis* Zhou et al. 2006 was sister to *H. guabangshanensis,* and *H. chinensis* was sister to *H. yiwuensis.* In the study of Li et al. (2011), *H. chinensis* formed a sister group with *H. maoershanensis* before they shared a common ancestor with *H. guabangshanensis*. Future studies aimed at elucidating these phylogenetic issues should incorporate more extensive taxon sampling and the addition of nuclear data, which may enhance phylogenetic accuracy.

In conclusion, this study presented the complete mitogenome of *H. bambusicolus* and inferred its phylogenetic position based on the concatenated nucleotide sequences of two rRNAs and 13 PCGs. The results of this study enrich the available genomic databases for *Hynobius* genus, which can be useful for future studies focused on evolution and conservation.

## Supplementary Material

Supplementary material revision.docx

## Data Availability

The mitochondrial genome sequence in this study is openly available at GenBank (https://www.ncbi.nlm.nih.gov/) under the accession number PQ220620. The associated BioProject, SRA, and BioSample numbers are PRJNA1187858, SRR31395777, and SAMN44819683, respectively.
