# Supplementary material for: Characterization of the complete mitochondrial genome of Hynobius bambusicolus Wang, Othman, Qiu and Borzée, 2023 (Amphibia, Caudata, Hynobiidae) and its phylogenetic implications
Source: Mitochondrial DNA B Resour. 2025 Mar 10;10(4):278–82. doi: 10.1080/23802359.2025.2475845 (PMC11894738; doi:10.1080/23802359.2025.2475845)
Supplement: Supplementary material revision.docx [file TMDN_A_2475845_SM8660.docx]

**Characterization of the complete mitochondrial genome of *Hynobius bambusicolus* and its phylogenetic implications**

Yanpin Huang1, Helin Wang1, Haoran Luo1, Honghui Zhong2, Qingxian Lin1* Xiaoping Zhou1*

1.Key Laboratory of the Ministry of Education for Coastal and Wetland Ecosystems, College of the Environment and Ecology, Xiamen University, Xiamen, Fujian Province, China

2. Meihua Mountain National Nature Reserve Administration, Longyan, Fujian Province, China


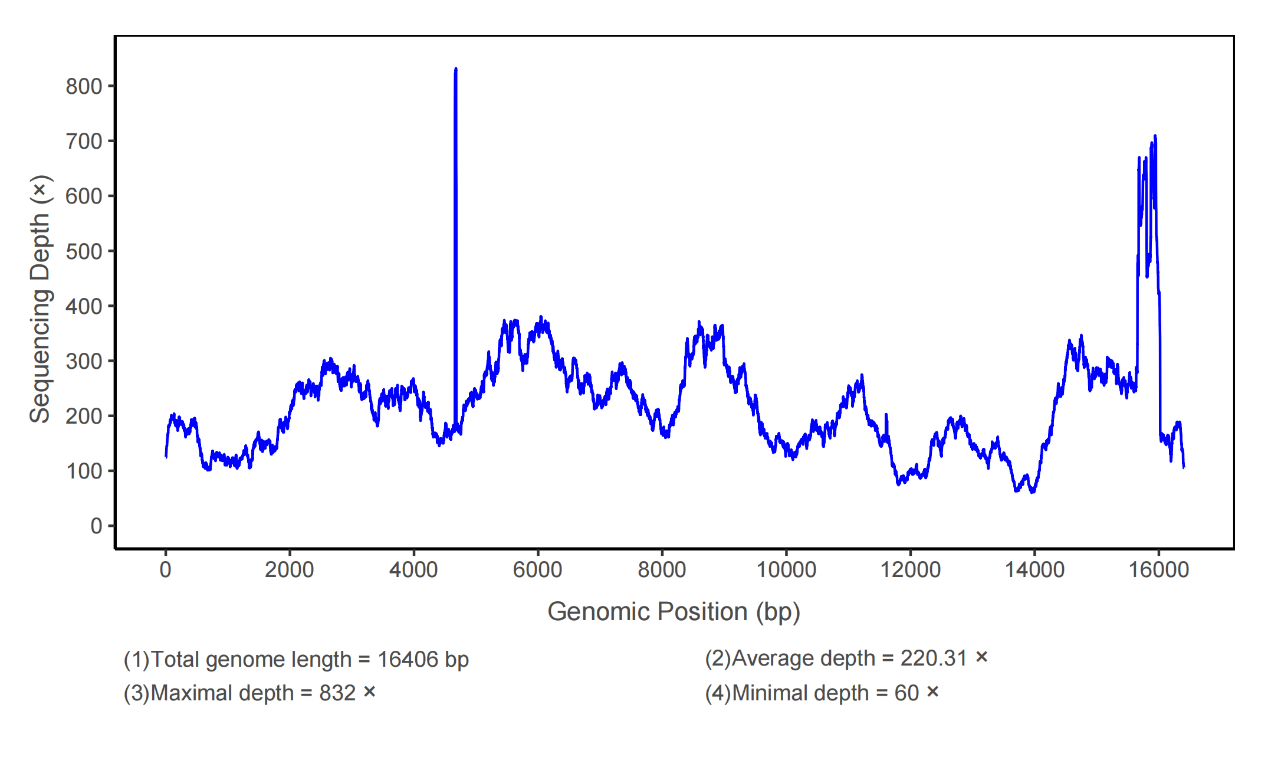


Figure S1 Sequencing depth and coverage map of the mitochondrial genome of *Hynobius bambusicolus*.

**Table S1**. Best sequence substitution model for each gene fragment using the Bayesian Information Criterion

| **Mitochondrial Gene Fragment** | **Best Sequence Substitution Model** |
| --- | --- |
| 12S rRNA | GTR+G |
| 16S rRNA | GTR+G+I |
| ND1 | HKY+G+I |
| ND2 | HKY+G+I |
| COX1 | HKY+G+I |
| COX2 | HKY+G+I |
| ATP8 | HKY+G |
| ATP6 | HKY+G |
| COX3 | GTR+G+I |
| ND3 | HKY+G+I |
| ND4L | HKY+G+I |
| ND4 | HKY+G+I |
| ND5 | GTR+G+I |
| ND6 | HKY+G+I |
| Cytb | GTR+G+I |
